# Population pharmacokinetic model and dosing nomogram for daptomycin in adult patients with serious Gram-positive infections: emphasizing the role of loading doses and renal function-based adjustment

**DOI:** 10.1128/aac.01532-25

**Published:** 2026-01-26

**Authors:** Petra Šubrtová, Petra Rozsívalová, Petra Halvová, Jana Maláková, Pavla Paterová, Lenka Ryšková, Josef Malý, Pavel Michálek, Ondřej Slanař, Martin Šíma

**Affiliations:** 1 Department of Clinical Pharmacy, Hospital Pharmacy, University Hospital Hradec Kralove 48234, Hradec Kralove, Czech Republic; 2 Department of Pharmacology, First Faculty of Medicine, Charles University and General University Hospital in Prague, Prague, Czech Republic; 3 Department of Social and Clinical Pharmacy, Faculty of Pharmacy in Hradec Kralove, Charles University 37740, Hradec Kralove, Czech Republic; 4 Department of Clinical Biochemistry and Diagnostics, University Hospital Hradec Kralove and Faculty of Medicine in Hradec Kralove, Charles University 37740, , Hradec Kralove, Czech Republic; 5 Department of Clinical Microbiology, University Hospital Hradec Kralove and Faculty of Medicine in Hradec Kralove, Charles University 37740, , Hradec Kralove, Czech Republic; 6 Department of Anaesthesiology, Resuscitation and Intensive Care Medicine, First Faculty of Medicine, Charles University and General University Hospital in Prague, Prague, Czech Republic; University of Houston, Houston, Texas, USA

**Keywords:** daptomycin, glomerular filtration rate, Monte Carlo simulation, nonlinear mixed-effects modeling, PK/PD target, therapeutic drug monitoring

## Abstract

Current recommendations for daptomycin dosing are based on body weight. However, it can be hypothesized that dosing based on renal function may improve the attainment of recommended pharmacokinetic (PK)/pharmacodynamic (PD) targets. The aim of this study was to develop a population pharmacokinetic model of daptomycin and propose an individualized dosing strategy to optimize target attainment. Therapeutic drug monitoring data from adult patients treated with daptomycin at a single center between 2022 and 2025 were analyzed using a nonlinear mixed-effects modeling. Monte Carlo simulations were then employed to identify the optimal dosing strategy that maximizes the probability of attaining the PK/PD target. A total of 143 daptomycin serum concentrations from 31 patients were included in the analysis. Estimated glomerular filtration rate (eGFR) was identified as the most predictive covariate for daptomycin pharmacokinetics. In a patient with an eGFR of 90 mL/min, the estimated volume of distribution and clearance of daptomycin were 11.47 L and 0.69 L/h, respectively. An eGFR-guided dosing nomogram was proposed, and simulation results demonstrated that this approach outperformed conventional weight-based dosing in achieving the PK/PD target. These findings support the use of an initial loading dose and individualized maintenance dosing based on eGFR to improve the efficacy and safety of daptomycin therapy.

## INTRODUCTION

Daptomycin is a cyclic lipopeptide antibiotic with potent activity against a broad spectrum of Gram-positive pathogens, including methicillin-resistant *Staphylococcus aureus* and vancomycin-resistant *Enterococcus* (1, 2). Owing to its unique mechanism of action, which involves calcium-dependent insertion into the bacterial cell membrane leading to rapid depolarization and cell death, daptomycin has become an important therapeutic option for complicated skin and soft tissue infections, bacteremia, and infective endocarditis (3). Despite its efficacy, the pharmacokinetic (PK) and pharmacodynamic (PD) variability of daptomycin among different patient populations poses a significant challenge to optimal dosing.

Daptomycin exhibits concentration-dependent bactericidal activity, and its efficacy is best correlated with the area under the concentration-time curve over 24 h to minimum inhibitory concentration (AUC₂₄/MIC) ratio (4, 5). However, this relationship is complicated by significant inter-individual variability in PK parameters, driven by patient-specific factors, such as renal function or disease severity (6). Current dosing recommendations are primarily based on total body weight, typically 4–12 mg/kg/day depending on the indication, type of pathogen, and pathogen susceptibility (7, 8). However, emerging evidence suggests that this weight-based approach may not adequately account for variability in renal function, the principal route of daptomycin elimination (6).

Approximately 50% of an administered daptomycin dose is excreted unchanged in the urine, underscoring the critical role of renal function in its disposition (7). Consequently, the estimated glomerular filtration rate (eGFR), as a marker of renal function, may serve as a more precise predictor of daptomycin clearance than body weight alone. This fact raises important questions regarding the adequacy of current dosing paradigms, particularly in patients at the extremes of renal function or body habitus. Population PK modeling provides a powerful tool for quantifying and simulating the influence of covariates, such as eGFR and body weight on daptomycin exposure, enabling individualized dose optimization. To date, several population PK models of daptomycin have been published, highlighting eGFR as a key covariate, while factors such as body weight, temperature, or disease severity have shown only negligible influence on the achievement of target daptomycin levels (9, 10). Moreover, while therapeutic drug monitoring (TDM) is not routinely employed for daptomycin, model-informed precision dosing strategies based on population PK models could bridge this gap and increase the proportion of patients achieving PK/PD targets.

Our primary hypothesis is that normalizing dosing to renal function rather than body weight will lead to improved attainment of the recommended PK/PD target. The current study aims to use available TDM data to develop a population pharmacokinetic model of daptomycin in adult patients with serious Gram-positive bacterial infections. Through simulation and probability of target attainment (PTA) analysis, we further explore how such an approach could inform individualized dosing strategies and potentially enhance clinical outcomes.

## MATERIALS AND METHODS

### Study design

A retrospective, single-center, cross-sectional study was performed in adult patients with serious Gram-positive bacterial infections treated with intravenous daptomycin admitted to mixed wards of the University Hospital Hradec Kralove from May 2022 to July 2025. Patients aged ≥18 years, receiving intravenous daptomycin, and having at least one measured daptomycin serum level as a part of a routine TDM procedure were eligible for inclusion in the pharmacokinetic analysis. Patients receiving renal replacement support were excluded from the analysis. The choice of a dosing regimen was entirely at the discretion of the attending physician in cooperation with the clinical microbiologist.

### Data retrieval

Clinical records of all patients meeting the inclusion criteria were reviewed to collect their demographic, laboratory, and clinical data. Du Bois’ body surface area, ideal body weight, adjusted body weight, and lean body mass were calculated using standard formulas (11). Creatinine-based eGFR was estimated according to the Chronic Kidney Disease Epidemiology Collaboration (CKD-EPI) 2012 and 2021 formulas for each patient (12, 13). Creatinine clearance was estimated using the Cockroft-Gault equation (14). Creatine kinase levels >3 ukat/L in females and >3.3 ukat/L in males were considered elevated. Similarly, myoglobin levels >68 μg/L in females and >86 μg/L in males were considered elevated. Daptomycin dosing regimen, including times of administration and infusion rates, and TDM data, including sampling times, were collected. If available, the MIC value of daptomycin for the isolated bacterial strain was also recorded.

### Bioanalytical and microbiological assay

Daptomycin serum concentrations were determined at the Department of Clinical Biochemistry and Diagnostics of University Hospital Hradec Kralove using a previously published ultra-performance liquid chromatography-tandem mass spectrometry method with some modifications (15, 16). After protein precipitation with acetonitrile, separation of analytes was realized on a Kinetex 2.6 µm HS F5 column using a mobile phase consisting of 10 mM ammonium formate buffer and acetonitrile with 0.1% formic acid. Analyses were performed on the Agilent Infinity 1290 UPLC system coupled with the Agilent 6470 Triple Quadrupole mass spectrometer. Detection was performed using an electrospray ionization technique. Monitored MS/MS transitions in the positive ion mode were m/z 811.1 > m/z 159 for daptomycin and m/z 609.4 > m/z 195 for reserpine (the internal standard). Validation experiments confirmed the suitability of the bioanalytical method. The calibration range and linearity were established from 0.32 mg/L (LLOQ) to 127.7 mg/L. Intra-day and inter-day precisions, expressed as coefficients of variation, ranged from 1.48% to 4.55% and from 2.06% to 6.31%, respectively. Accuracy (bias) ranged from −8.4% to 10.9% for intra-day measurements and from 0.02% to 3.4% for inter-day measurements.

All microbiological samples were processed at the Department of Clinical Microbiology of the University Hospital Hradec Kralove. All pathogens were identified by MALDI-TOF MS (Bruker Daltonics GmbH, Bremen, Germany). Antibiotic susceptibility examination was performed by the E-test (bioMérieux, Marcy l’Etoile, France) in Mueller-Hinton agar (Thermo Fisher Scientific, Basingstoke, UK), and the results were interpreted according to the European Committee on Antimicrobial Susceptibility Testing (17).

### Population pharmacokinetic analysis

The nonlinear mixed-effects modeling software Monolix Suite version 2024R1 (Lixoft SAS, Antony, France), implementing the stochastic approximation expectation-maximization algorithm, was used to analyze daptomycin serum concentration-time data. One- and two-compartment models with first-order or saturation kinetics of elimination were tested as structural models. The model parameters were assumed to be log-normally distributed. For each pharmacokinetic parameter, log-normally distributed inter-individual variability terms with estimated variance were tested. To describe the residual variability, constant, proportional, or combined error models were tested. The best structural model was selected based on the following predetermined criteria: objective function value (OFV), Akaike information criterion and Bayesian information criterion differences, visual inspection of the goodness-of-fit (GOF) plots, shrinkage in distribution of the individual parameters, and low relative standard errors (R.S.E.) of the parameter estimates.

In the second step, the effects of different variables on the base structural model were assessed; age, height, body weight, ideal body weight, adjusted body weight, lean body mass, and body surface area were tested as continuous covariates, while sex was tested as a categorical covariate of pharmacokinetic parameters. Time-varying covariates, including serum creatinine, eGFR estimated according to both 2012 and 2021 CKD-EPI formula, creatinine CL estimated according to the Cockroft-Gault formula, and serum urea, were tested by incorporating them as regressors. Both the model in which the regressor influences total CL and the model in which the regressor affects only renal CL while non-renal CL remains unaffected were tested. The potential covariates were preliminarily identified using a graphical assessment and univariate association using Pearson’s correlation test of the effects of covariates on estimated pharmacokinetic parameters. Significant covariates from this process were further confirmed or rejected using a manual stepwise forward inclusion and backward elimination procedure. For model selection, a decrease in OFV of >3.84 points between nested models (χ^2^-test, α = 0.05 for 1 degree of freedom) was considered statistically significant. In the backward elimination, covariates were retained in the model if the difference in OFV was greater than 6.64 points between nested models (*P* < 0.01). Additional criteria for model selection were reasonably low R.S.E. values of the estimates of model parameters (˂50%), physiological plausibility of the obtained parameter values and the covariates found, and absence of bias in GOF plots.

The evaluation of the final model was based on individual model fitting curves, GOF plots (observations versus individual and population predictions), and internal validation using visual predictive check (VPC), displaying both time after the last dose and the eGFR trajectories for the 10th, 50th, and 90th percentiles of the simulated profiles (1,000 replicates) overlaid with the observed data. The accuracy of the final model was further evaluated using a bootstrap analysis. In this procedure, 500 replicates of the original data were generated, and the parameter estimates for each of the 500 samples were re-estimated in the final model. The median and 95% confidence intervals (CIs) obtained for each of the parameters estimated for bootstrap samples were compared with the estimates in the final model.

### Monte Carlo simulations

The final population pharmacokinetic model for daptomycin (including residual unexplained variability) was imported to Simulx software 2024R1 (Lixoft SAS, Antony, France) to perform Monte Carlo simulations (500 replicates of the original data set) of the theoretical distribution of concentration-time profiles across the population under different dosing regimens—standard approved daptomycin dosage of 6 mg/kg (administered via 30-min infusion) once daily and the real dosage used in our study population (7–13 mg/kg, every 24 or 48 h via 30- or 60-min infusion). The ratio of AUC_24_/MIC ≥666 was considered as a PK/PD target for efficacy, while trough concentration (C_min_) ˃24 mg/L was considered as a threshold for toxicity (5). The PTA for both efficacy and toxicity was calculated for both dosing regimens, considering MIC values of 0.5 mg/L (the median and mode in our population) and 1 mg/L (the daptomycin breakpoint value for *Staphylococcus* spp.) (17). Subsequently, administration of daptomycin dosing scaled by eGFR (as found covariate of daptomycin CL) was simulated to maximize the PTA for efficacy and minimize the PTA for toxicity. For this purpose, the relationship between eGFR and daptomycin CL, described in the final population pharmacokinetic model, was converted into a dosing nomogram for the determination of the optimal daptomycin daily dose based on the patient’s eGFR. Since the simulations showed a lower PTA for efficacy on the first day of therapy (before the steady-state achievement), we also proposed the administration of a loading dose calculated as the product of the maintenance dose (based on eGFR) and the ratio of AUC_24_ between the first and seventh day of therapy (expressing the degree of accumulation).

### Individual data processing and statistics

Descriptive parameters—median, interquartile range (IQR), and 95% CIs for medians were calculated using GraphPad Prism 8.2.1 software (GraphPad Inc., La Jolla, USA). Individual values of the AUC_24_ on the seventh day of daptomycin therapy at an approved dosing of 6 mg/kg every 24 h were estimated as the Empirical Bayes Estimates in Simulx software 2024R1, and then its relationship with C_min_ was evaluated using the linear regression model in GraphPad Prism 8.2.1 software. The Empirical Bayes Estimates were also used to calculate the cumulative exposure to daptomycin during the first 3 days of therapy (AUC_072h_) at the real dosage used in our study population. These values were then compared between patients with and without elevated creatine kinase and myoglobin levels. Mann-Whitney test in GraphPad Prism 8.2.1 software was used for this comparison, and *P* levels <0.05 were considered statistically significant.

## RESULTS

### Study population

Thirty-one patients were eligible for inclusion in pharmacokinetic analysis. The most frequent diagnoses for antibiotic treatment were infective endocarditis (*n* = 14; 45.2%), bone and joint infections (*n* = 6; 19.4%), sepsis (*n* = 3; 9.7%), and catheter-associated infections (*n* = 3; 9.7%). The most common causative agent of infections was *Staphylococcus* spp. (*n* = 21; 67.7%), *Enterococcus* spp. (*n* = 4; 12.9%), and *Corynebacterium* spp. (*n* = 2; 6.5%). The MIC value for daptomycin was determined in 19 cases. The median (range) and mode of MIC were 0.5 (0.016–1.5) mg/L and 0.5 mg/L, respectively. Demographic, laboratory, and clinical data of patients are summarized in Table 1.

**TABLE 1:** Demographic, laboratory, and clinical data of the patients^*c*^^,^^*d*^

Feature	Median (IQR) [range] or N (%)
Sex M/F	24 (77.4)/7 (22.6)
Age (years)	63 (53–75) [29–93]
Height (cm)	172 (170–180) [158–191]
Body weight (kg)	84 (73–92) [48–124]
Ideal body weight (kg)	68 (61–75) [51–85]
Adjusted body weight (kg)	73 (67–82) [48–98]
Lean body mass (kg)	59 (53–66) [41–81]
Body surface area (m^2^)	1.98 (1.86–2.12) [1.50–2.47]
Serum creatinine (µmol/L)	90 (66–117) [36–324]
eGFR CKD-EPI 2012 (mL/min)	73.5 (55.0–102.7) [14.9–132.1]
eGFR CKD-EPI 2021 (mL/min)	81.6 (58.9–98.9) [16.3–130.1]
CLcr Cockroft-Gault (mL/min)	54.2 (38.1–86.8) [15.2–176.0]
Urea (mmol/L)	7.1 (4.6–13.7) [2.2–29.7]
C-reactive protein (mg/L)	112 (32–154) [0.2–371]
Creatine kinase (ukat/L)^*a*^	0.56 (0.40–0.80) [0.16–4.55]
Myoglobin (μg/L)^*b*^	37.6 (21.9–65.73) [21.0–192.7]

^*a*^ Measured at the start of daptomycin therapy in 24 patients.

^*b*^ Measured at the start of daptomycin therapy in 17 patients.

^*c*^ Data are expressed as median (IQR) [range] or N (%).

^*d*^ Values at the start of daptomycin therapy.

Daptomycin was administered as a part of routine clinical practice in a median (min-max) dose of 750 (350–1,000) mg, corresponding with a body weight-normalized dose of 10 (5–13) mg/kg, every 24 or 48 h via 30- or 60-min intravenous infusion. This initial dosage was further adjusted based on TDM.

**Fig 1:**
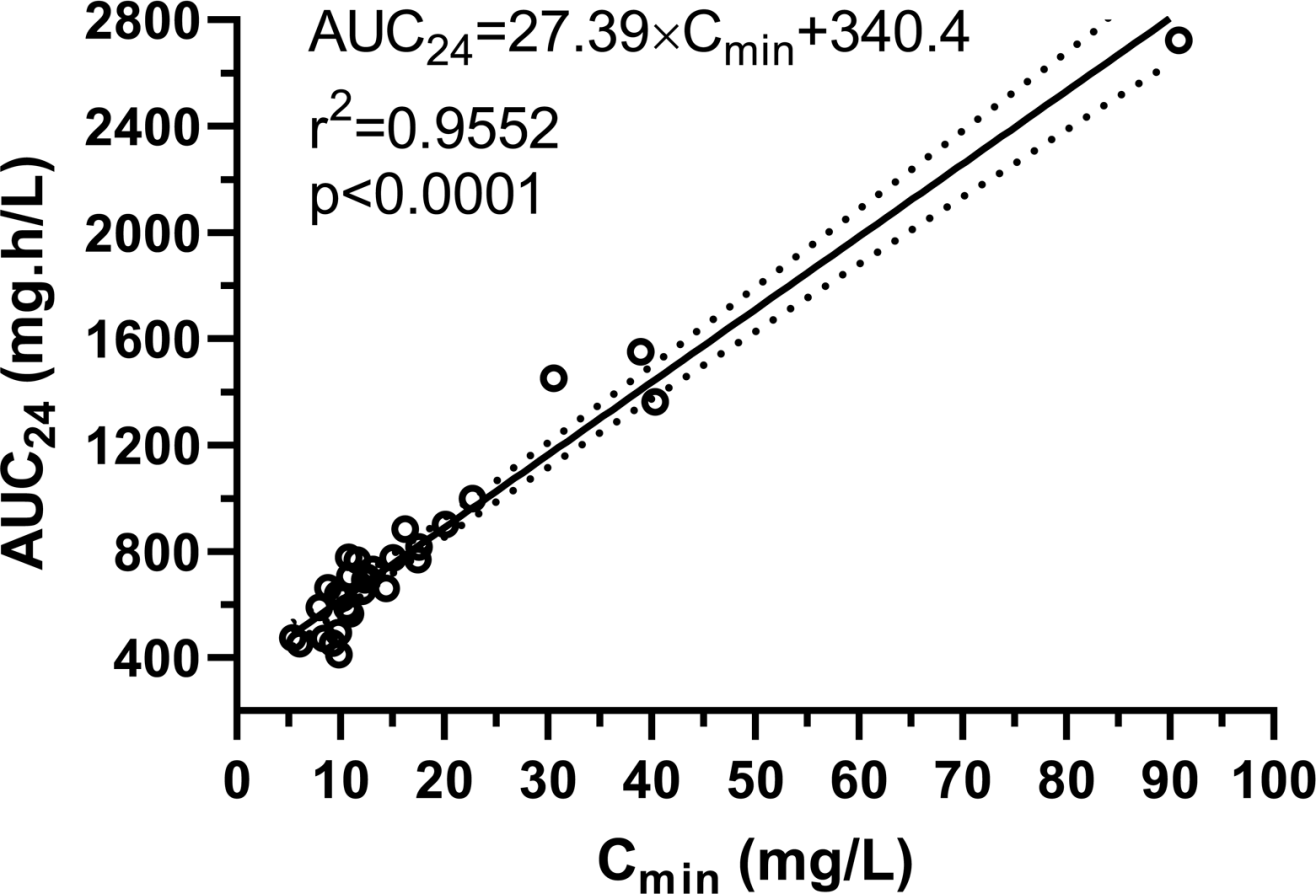
Relationship between daptomycin trough (C_min_) levels at steady state (seventh day of therapy) and daptomycin exposure (AUC_24_) based on simulated individual daptomycin pharmacokinetic profiles using the Empirical Bayes Estimates.

A total of 143 measured serum concentrations of daptomycin (1–19 per patient, average 4.6 per patient) were included in the analysis. Eighty samples were taken as trough levels (sampling 1 h before the next dose) and 63 samples as peak levels (sampling 2 h after the start of the infusion, i.e., 1 or 1.5 h after the end of the infusion). In two (6.5%) patients, only trough concentrations were measured, while in 29 (93.5%) patients, both trough and peak levels were measured. Simulation based on the Empirical Bayes Estimates of individual daptomycin pharmacokinetic profiles showed an excellent predictive performance of steady-state (seventh day) daptomycin trough (C_min_) levels for estimation of total daptomycin exposure (AUC_24_) (Fig. 1).

Creatine kinase and myoglobin levels were elevated in six and eight patients, respectively. There was no significant difference in the first 3-day cumulative exposure to daptomycin (AUC_072h_) between patients with and without creatinine kinase elevation (median AUC_072h_ of 2,909 vs 3,052 mg·h/L, *P* = 0.9031). Similarly, the difference in daptomycin AUC_072h_ between patients with and without elevated myoglobin did not reach statistical significance (median AUC_072h_ of 3,095 vs 3,013 mg·h/L, *P* = 0.3388).

### Population pharmacokinetic analysis

**Fig 2:**
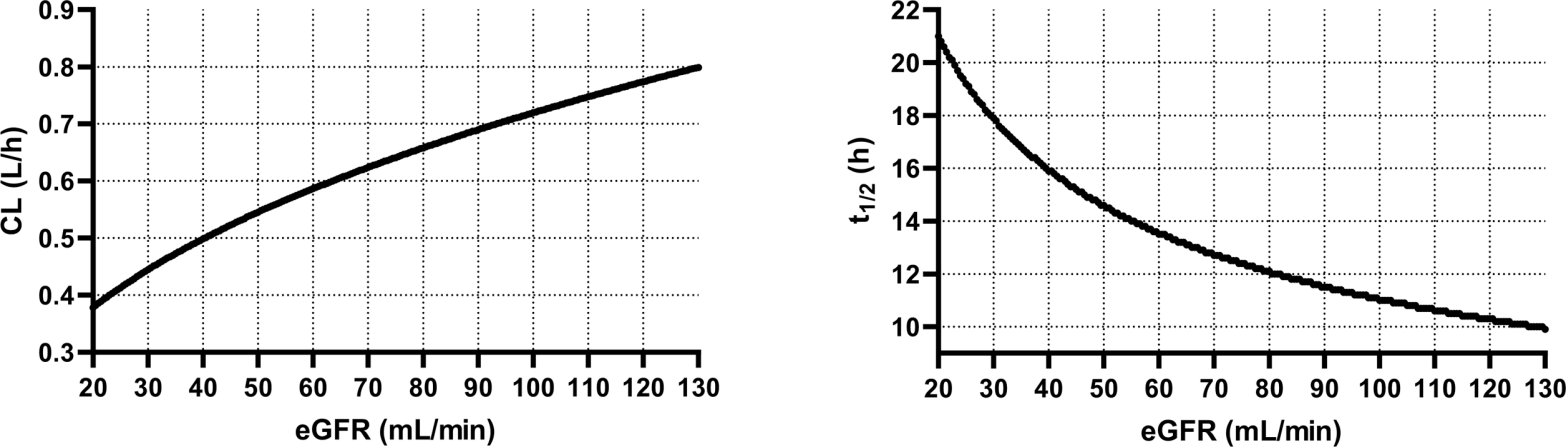
Relationship between daptomycin CL and t_1/2_ and the patient’s eGFR estimated using the CKD-EPI_2021_ formula.

A one-compartmental model with linear elimination kinetics and a proportional error model was selected as the best structural and error model to describe the daptomycin concentration-time data and explain the residual variability, respectively. The pharmacokinetic model was parametrized in terms of the volume of distribution (Vd) and clearance (CL). Based on the covariate model analysis of all the tested variables, the most appropriate covariate was CKD-EPI_2021_ eGFR for daptomycin CL (Fig. S1). A model in which eGFR, as a time-varying covariate, influences total CL was found to be more suitable. The summary of the model-building process is provided in Table S1. The appropriateness of eGFR as a covariate of daptomycin CL was confirmed based on the decrease in OFV of 28.74 points and the physiological plausibility. The other tested variables exerted no influence on daptomycin pharmacokinetics. The final equations for the description of the relationships between the daptomycin pharmacokinetic parameters and their covariates, the population pharmacokinetic estimates, and bootstrap results from the final population model are summarized in Table 2. In the final model, for an individual with normal renal function status (eGFR = 90 mL/min), daptomycin Vd and CL were 11.47 L and 0.69 L/h, respectively, which corresponds to the elimination half-life (t_1/2_) of 11.5 h. For example, in a patient with severe renal insufficiency with an eGFR of 30 mL/min, the CL of daptomycin will decrease to 0.44 L/h, which corresponds to a t_1/2_ of 18.1 h. On the contrary, a patient with augmented renal clearance of 130 mL/min will have a daptomycin CL of 0.8 L/h, and thus a t_1/2_ of 9.9 h. The relationship between CL and t_1/2_ of daptomycin and eGFR is shown in Fig. 2.

**TABLE 2:** Estimates of the final daptomycin population pharmacokinetic model and the bootstrap results based on 500 simulations^*a*^

	Final model		Bootstrap analysis
Parameter	Estimate	R.S.E. (%)	Median (95% CI)
Fixed effects			
*Vd = Vd_pop* *CL = CL_pop × (eGFR/90)^β_CL_eGFR^*			
Vd_pop (L)	11.47	6.88	11.56 (11.45–11.65)
CL_pop (L/h)	0.69	5.13	0.70 (0.69–0.70)
β_CL_eGFR	0.40	32.40	0.41 (0.39–0.43)
Between subject variability (%)			
Vd	18.0	29.8	16.0 (15.0–17.0)
CL	20.0	22.3	18.2 (17.0–18.0)
β_CL_eGFR	39.0	29.8	41.0 (40.0–43.0)
Error model parameter			
Proportional	0.28	7.72	0.28 (0.28–0.29

^*a*^ Vd, volume of distribution; CL, clearance; pop, typical value of parameter; β_CL_eGFR, effect of eGFR (mL/min) on CL; eGFR, glomerular filtration rate estimated according to CKD-EPI_2021_ formula; CI, confidence interval; RSE, relative standard error.

Figure S2 shows the individual-to-population estimates of CL versus eGFR for both the base structural model and the final covariate model. As expected, the base model exhibits a clear trend between eGFR and the individual deviations in CL, whereas this trend is eliminated in the final model after inclusion of eGFR as a covariate. The diagnostic GOF plots (Fig. S3) for the final covariate model indicated a good fit of the model to daptomycin pharmacokinetics in the targeted population. The VPC comparing observed daptomycin concentrations with model-predicted percentiles over time after the last dose (Fig. S4) shows that the population PK model adequately captures both the central tendency and variability of the data. Observed medians and prediction intervals fall largely within the simulated confidence bands across the full post-dose interval, indicating no major time-dependent model misspecification. The VPC stratified by eGFR (Fig. S5) demonstrates good predictive performance of the model across the range of renal function. The model appropriately reproduces the trends in observed concentrations and their variability, with observed percentiles aligning well with the simulated prediction intervals, supporting the suitability of the renal function covariate relationship. As shown in Table 2, the R.S.E. (maximum 32.4%) revealed that all the pharmacokinetic parameters in the model were estimated precisely. All the median parameter values of the bootstrapping replicates were close (maximal difference of 11%) to the final estimates of all parameters, confirming the estimated values.

### Monte Carlo simulations

**Fig 3:**
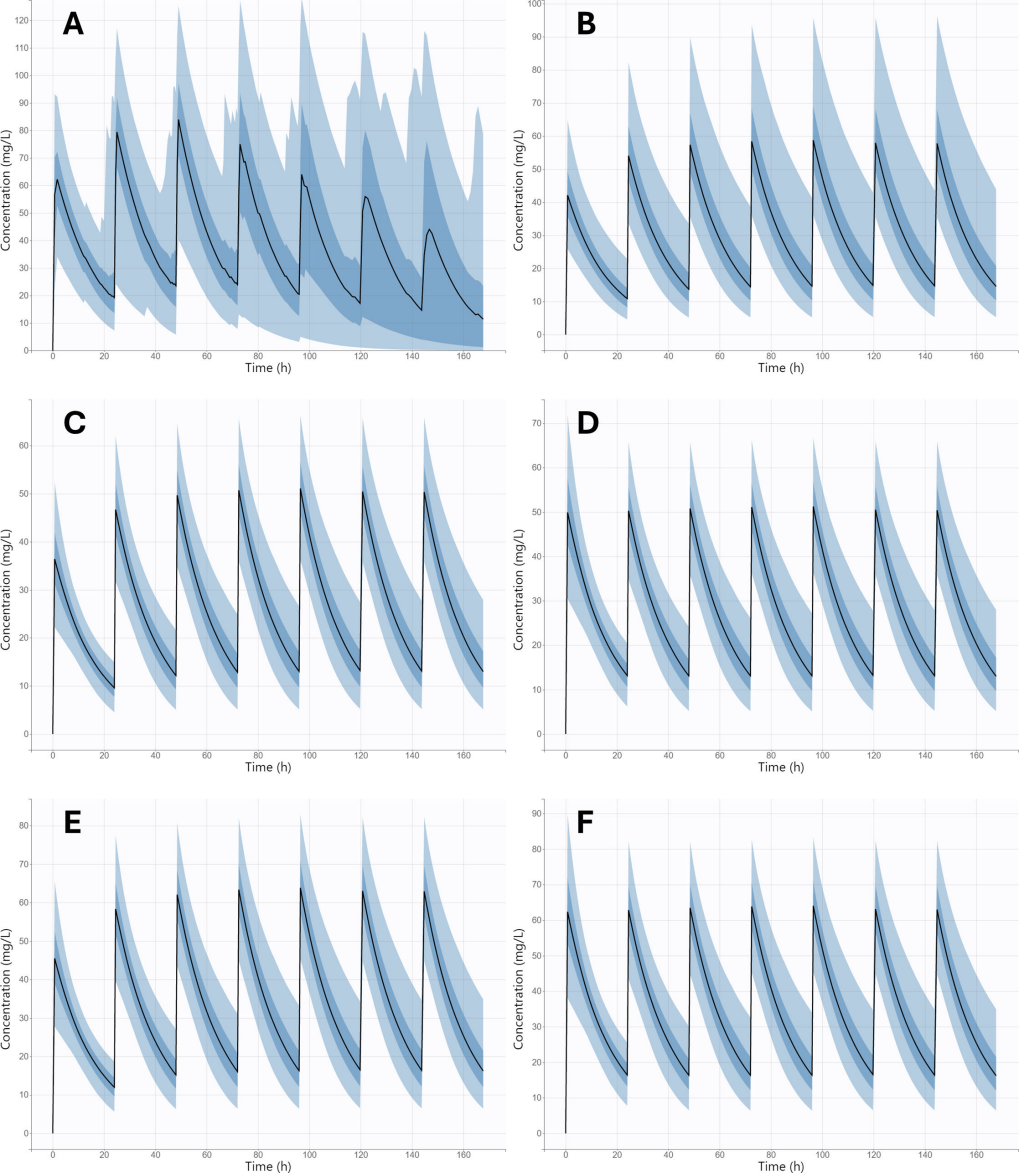
Monte Carlo simulation of daptomycin serum concentration-time profiles following intravenous administration: (**A**) real dosages used in our study population (5–13 mg/kg, every 24 or 48 h via 30- or 60-min infusion); (**B**) standard approved daptomycin dosage of 6 mg/kg (administered via 30-min infusion) once daily; (**C**) eGFR-based maintenance dose according to the nomogram for MIC = 0.5 mg/L, once daily via 30-min infusion; (**D**) loading dose, followed by eGFR-based maintenance dose according to the nomogram for MIC = 0.5 mg/L, once daily via 30-min infusion; (**E**) eGFR-based maintenance dose according to the nomogram for MIC = 1 mg/L, once daily via 30-min infusion; (**F**) loading dose, followed by eGFR-based maintenance dose according to the nomogram for MIC = 1 mg/L, once daily via 30-min infusion. The black line represents the median, and the four blue bands represent the percentiles (5%–27.5%, 27.5%–50%, 50%–72.5%, and 72.5%–95%) of the 90% simulated concentration distribution.

**Fig 4:**
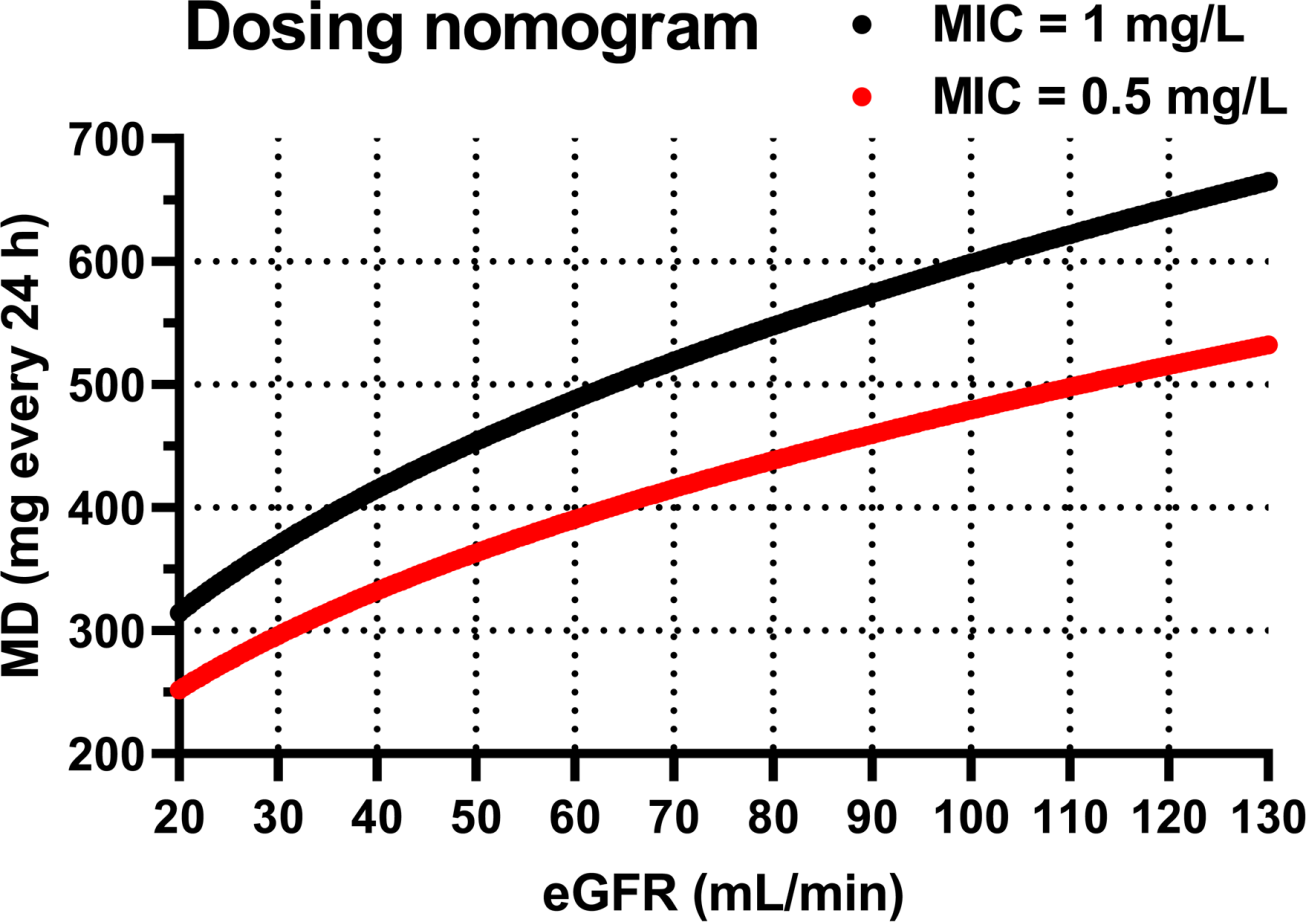
Nomogram for calculating the daptomycin maintenance dose (MD), administered once daily via 30-min intravenous infusion, based on the patient’s eGFR estimated using the CKD-EPI_2021_ formula, at MIC values of 0.5 mg/L (red curve) and 1 mg/L (black curve).

Figure 3 represents the theoretical distribution of the daptomycin serum concentration-time profiles at administration of four alternative dosing regimens. Firstly, daptomycin pharmacokinetic profiles at administration of the real dosage used in our study population (Fig. 3A) and at standard approved daptomycin dosage (Fig. 3B) were simulated. Subsequently, to maximize the PTA for efficacy (at MIC values of 0.5 and 1 mg/L) and minimize the PTA for toxicity, administration of dosing individualized according to the patient’s eGFR, as a covariate of daptomycin CL, was simulated (Fig. 3C and E). For this purpose, the relationship between eGFR and daptomycin CL (Fig. 2) was converted into an easy-to-use dosing nomogram for the determination of the optimal daptomycin daily dose based on the patient’s eGFR (Fig. 4). Since the simulations showed a lower PTA for efficacy on the first day of therapy (AUC_24_ was 1.36 times lower than at steady state), we also simulated the administration of dosage according to the nomogram with initial administration of a loading dose equal to 1.36 times the maintenance dose (Fig. 3D and F). Table 3 summarizes the PTA values for the PK/PD targets for efficacy (considering MIC values of 0.5 and 1 mg/L) and for the toxicity threshold, as well as the AUC_24_ values, at various daptomycin dosing regimens on the first and seventh day of therapy.

**TABLE 3:** PTA for PK/PD efficacy targets (considering MIC values of 0.5 and 1 mg/L), the toxicity threshold, and AUC_24_ values at various daptomycin dosing regimens on the first and seventh day of therapy^*b*^

Day	Parameter	Regimen					
		Real dosage used (intuitive dosing)	SmPC	eGFR-based nomogram (MIC = 1 mg/L)	eGFR-based nomogram + LD (MIC = 1 mg/L)	eGFR-based nomogram (MIC = 0.5 mg/L)	eGFR-based nomogram + LD (MIC = 0.5 mg/L)
1	PTA: AUC_24_ ≥ 333 mg·h/L (%)	100 (100–100)	96.8 (90.3–100)	100 (96.8–100)	100 (96.8–100)	96.8 (90.3–100)	100 (96.8–100)
	PTA: AUC_24_ ≥ 666 mg·h/L (%)	83.9 (77.4–87.1)	25.8 (16.1–32.3)	25.8 (12.9–38.7)	87.1 (77.4–96.8)	0 (0–6.5)	48.4 (35.5–61.3)
	PTA: C_min_ ˃ 24 mg/L (%)	35.5 (25.8–45.2)	3.2 (0–9.7)	0 (0–3.2)	6.45 (0–16.1)	0 (0–0)	0 (0–4.8)
	AUC_24_ (mg·h/L)	892.9 (832.6–957.5)	557.1 (519–598.1)	602.8 (567.9–636.9)	825.9 (778.1–872.5)	481.5 (455.5–510.4)	660.7 (622.3–699.3)
7^*a*^	PTA: AUC_24_ ≥ 333 mg·h/L (%)	100 (96.8–100)	100 (96.8–100)	100 (96.8–100)	100 (96.8–100)	100 (96.8–100)	100 (93.6–100)
	PTA: AUC_24_ ≥ 666 mg·h/L (%)	90.3 (83.9–96.8)	64.5 (51.6–74.2)	80.7 (67.7–90.3)	80.7 (67.7–90.3)	48.4 (35.5–62.9)	48.4 (33.9–64.5)
	PTA: C_min_ ˃ 24 mg/L (%)	48.4 (38.7–61.3)	19.4 (9.7–29)	19.4 (9.7–32.3)	19.4 (9.7–29)	8.1 (0–16.1)	9.7 (3.2–16.1)
	AUC_24_ (mg·h/L)	1,132.5 (1,038.2–1,253.1)	747.8 (670.6–834.3)	821 (751.6–906.2)	825.7 (754.8–906.6)	658.4 (601.8–717.5)	660.6 (606–719.4)
Median AUC_24_ ratio (day 7 vs day 1)		NA	1.34	1.36	1.0	1.37	1.0

^*a*^ For intuitive dosing, the PTA value from the third day of therapy is given (because the dosage was then adjusted based on TDM). NA, not applicable; AUC_24_, area under the concentration-time curve during a 24-h period; C_min_, minimum (trough) serum concentration; SmPC, summary of product characteristics; eGFR, estimated glomerular filtration rate; LD, loading dose.

^*b*^ Data except ratio are expressed as median (90% CI) from replicates.

## DISCUSSION

The conventional approach to daptomycin dosing is based primarily on body weight—typically expressed in mg per kg of total body weight (3, 7). While this strategy is simple and widely used, it assumes that body weight is the main determinant of drug exposure. However, if daptomycin CL is more strongly influenced by renal function (as can be expected in a drug that is mainly eliminated by the kidneys), weight‐based regimens may lead to suboptimal exposure (either under‐ or over‐exposure), especially in patients with impaired or augmented renal function. Through the routine use of TDM for daptomycin in our hospital, we had the opportunity to develop a population PK model for this drug and use model-based simulations to test our hypothesis regarding dosage optimization.

In our population PK analysis, eGFR emerged as the sole statistically significant covariate for daptomycin clearance. This finding aligns with literature indicating that renal function is a major driver of variability in exposure to daptomycin, as a drug primarily eliminated by the kidneys (9, 10, 18). The values of pharmacokinetic parameters (both Vd and CL) correspond to the values given in the text approved by regulatory authorities (7). In the scientific literature, daptomycin CL in patients with preserved renal function is often reported around 0.8 L/h (9, 10), slightly higher than the value observed in our study (0.69 L/h). This difference may be attributable to subtle variations between cohorts, such as differences in clinical condition (19). Some studies reporting higher daptomycin CL in patients with normal renal function define this subpopulation categorically—for example, as those with an eGFR above 80 mL/min (20). Consequently, this subpopulation may include patients with augmented renal clearance, which can result in higher observed daptomycin CL values.

On the contrary, body-size descriptors did not correlate significantly with either Vd or CL. The study cohort, although modest in size (*n* = 31), exhibited a wide range of body-size characteristics (body weight 48–124 kg; height 158–191 cm). Such substantial variability would be expected to reveal any clinically meaningful effect of body size on PK parameters, had one been present. Neither exploratory covariate-parameter relationships nor formal covariate modeling indicated any trend between body-size descriptors (body weight, height, adjusted body weight, lean body mass, body surface area) and daptomycin CL or Vd. This suggests that the absence of a detected effect is unlikely to be solely attributable to limited sample size, but rather reflects a genuinely minor or negligible influence of body-size metrics in this population. Moreover, body size descriptors are commonly associated with the prediction of Vd rather than CL. Since Vd primarily influences the peak concentration at the end of the distribution phase, whereas AUC-targeted dosing is mainly determined by CL, the lack of observed body size covariates in our study is unlikely to affect the PK/PD target attainment. Accordingly, our dosing recommendation is based on eGFR as a covariate of CL, which remains the key determinant for achieving the desired exposure.

Several PK/PD targets for daptomycin have been proposed (21). For efficacy, we applied the AUC_24_/MIC >666 threshold, which has been consistently linked to microbiological and clinical success in multiple studies evaluating *Staphylococcus aureus* infections and is frequently used in population pharmacokinetic modeling and dosing-optimization research (5, 6). For safety, we used a C_min_ >24 mg/L cut-off, as this value has been repeatedly associated with an increased risk of creatine phosphokinase elevation and daptomycin-related musculoskeletal toxicity across several independent cohorts (21, 22). Among the available toxicity markers, this threshold has shown the most reproducible and clinically meaningful association with adverse outcomes. Although alternative PK/PD targets have been suggested, we selected these two because they are the most robustly supported and widely applied in previous PK/PD and clinical investigations, thereby facilitating comparability with existing literature.

Given that the only significant covariate we identified was eGFR, we used Monte Carlo simulations to compare standard weight‐based dosing regimens against newly proposed dosing adjusted by eGFR. We evaluated outcomes in terms of the PTA for the PK/PD efficacy targets of AUC_24_ ≥666 mg·h/L (for a MIC of 1 mg/L) and AUC_24_ ≥333 mg·h/L (for a MIC of 0.5 mg/L), while simultaneously limiting the risk of toxicity, as indicated by a trough concentration C_min_ >24 mg/L. When designing the eGFR-based dosing nomogram (Fig. 4), we needed to target the midpoint of a defined therapeutic range. Therefore, it was necessary to unify the parameters for efficacy and toxicity. We therefore converted the C_min_ value of 24 mg/L to an AUC_24_ value of 998 mg·h/L, which corresponds to the relationship between C_min_ and AUC_24_ (Fig. 1). After all, it is likely that the toxicity of daptomycin is actually proportional to its total exposure, and C_min_ is only a surrogate marker of this exposure (22). Thus, as the midpoint of the therapeutic ranges of 666–998 and 333–998 mg·h/L, we targeted AUC_24_ values of 832 and 665.5 mg·h/L, respectively. Our simulations showed that eGFR‐based dosage adjustment consistently resulted in improved achievement of daptomycin exposures within the therapeutic range (Table 3), as also documented by the narrowed distribution of pharmacokinetic profiles between the 5th and 95th percentiles in the population (Fig. 3). Compared to body weight-based dosing according to the SmPC, dosing according to the proposed eGFR-based nomogram resulted in a significantly higher proportion of patients meeting the PK/PD target for efficacy (80.7% vs 64.5% at MIC = 1 mg/L). On the contrary, when using high-dose regimens normalized by weight, as recommended by some studies for the treatment of severe infections (4), a very high proportion of patients exceeded the toxicity threshold compared to the use of the eGFR-based nomogram (48.4% vs 19.4%). Using weight‐based dosing alone leads to insufficient exposure, especially in patients with high eGFR (augmented renal clearance), or, conversely, to excessive accumulation in patients with reduced eGFR (renal insufficiency), problems that dosing based on eGFR can help to avoid.

Another point revealed by our modeling is that a loading dose allows immediate achievement of therapeutic exposure on day one, rather than waiting for several doses to accumulate exposure. This may be particularly important in severe infections, where delays in reaching the PK/PD target could compromise efficacy. Based on the observed accumulation ratio of daptomycin derived from its pharmacokinetic parameters, we recommend administering a loading dose equal to 1.36 times the maintenance dose according to the eGFR-based nomogram.

It should be noted that the proposed dosage nomograms are valid only under the assumptions of MIC ≤1 mg/L (black curve in Fig. 4) and MIC ≤0.5 mg/L (red curve in Fig. 4), which correspond to the daptomycin breakpoint value for *Staphylococcus* spp. and the most frequent MIC value, respectively. If targeting a higher MIC value, dose adjustments would be necessary. However, in our study, both the median and mode MIC values were 0.5 mg/L, and the MIC value of 1 mg/L was exceeded in only one case, where *Enterococcus faecium* was identified as the infectious agent, with an MIC of 1.5 mg/L.

Due to the retrospective nature of our study, we did not measure concentrations of unbound daptomycin. Although daptomycin binding to plasma proteins was described to be high (~92%), its affinity for plasma proteins seems to be weaker than its irreversible interaction with the bacterial membrane, which leads to a significantly higher availability than would be expected based on its level of protein binding (23). Although the efficacy of antibiotic drugs is more closely related to the unbound concentration rather than the total drug levels in the blood, the clinical PK/PD target for daptomycin efficacy relies on total concentrations (6). Accordingly, the position paper specifies the PK/PD target based on total daptomycin concentrations as AUC_24_/MIC, in contrast to, for example, beta-lactams or colistin, where fT>MIC or fAUC_24_/MIC is reported (5).

### Conclusion

The developed population pharmacokinetic model of daptomycin in adult patients with severe Gram-positive infections identified eGFR as the most significant covariate influencing and predicting daptomycin pharmacokinetics. Utilizing this insight, a dosing nomogram guided by eGFR was proposed to optimize daptomycin therapy. Simulation results demonstrated that this approach outperforms the conventional weight-based dosing in achieving the PK/PD target. Moreover, model-based simulations supported the benefit of initiating treatment with a loading dose equal to 1.42 times the maintenance dose to ensure early therapeutic exposure. These findings provide a rationale for incorporating renal function into routine dosing strategies to enhance the efficacy and safety of daptomycin treatment.

## Data Availability

The data that support the findings of this study are available from the corresponding author upon reasonable request.

## SUPPLEMENTAL MATERIAL

Supplemental material aac.01532-25-s0001.docx Table S1; Fig. S1 to S5.

ASM does not own the copyrights to Supplemental Material that may be linked to, or accessed through, an article. The authors have granted ASM a non-exclusive, world-wide license to publish the Supplemental Material files. Please contact the corresponding author directly for reuse.
