# Supplementary material for: Population pharmacokinetic model and dosing nomogram for daptomycin in adult patients with serious Gram-positive infections: emphasizing the role of loading doses and renal function-based adjustment
Source: Antimicrob Agents Chemother. 2026 Jan 26;70(3):e01532-25. doi: 10.1128/aac.01532-25 (PMC12959151; doi:10.1128/aac.01532-25)

**Supplementary data**

**Population pharmacokinetic model and dosing nomogram for daptomycin in adult patients with serious Gram-positive infections: emphasizing the role of loading doses and renal function-based adjustment**

**Table S1.** The summary of the model building process.

|  | OFV | AIC | BIC | Max R.S.E. (%) | Max shrinkage of individual parameters (%) |
| --- | --- | --- | --- | --- | --- |
| ***Structural model*** | | | | | |
| **1-compartment+1^st^ order kinetics** | 1201.98 | 1211.98 | 1219.15 | 28.8 | 48.6 |
| 2-compartment+1^st^ order kinetics | 1185.58 | 1203.58 | 1216.49 | 7.76×e^17^ | 100.0 |
| 1-comp+ saturation kinetics | 1202.01 | 1216.01 | 1226.05 | 85.8 | 65.3 |
| ***Error model*** | | | | | |
| **Proportional** | 1201.98 | 1211.98 | 1219.15 | 28.8 | 48.6 |
| Constant | 1269.52 | 1279.52 | 1286.69 | 24.0 | 41.0 |
| Combined | 1202.05 | 1214.05 | 1222.66 | 1.01×e^3^ | 48.3 |
| ***Time-varying covariate model*** | | | | | |
| CL = CLnr_pop + CLr_pop × (eGFR2021/90)^β_CL_eGFR2021^ | 1178.16 | 1196.16 | 1209.06 | 1.37×e^8^ | 100.0 |
| **CL = CL_pop × (eGFR2021/90)^β_CL_eGFR2021^** | 1173.24 | 1187.24 | 1197.28 | 32.4 | 46.6 |

*The selected model at each step is highlighted in bold.*

*CLnr – non-renal clearance; CLr – renal clearance; pop – typical value of parameter; β_CL_eGFR – effect of eGFR (mL/min) on CL; R.S.E. – relative standard error; OFV –objective function value; AIC – Akaike information criterion; BIC – Bayesian information criterion.*

**Figure S1.** Conditional mode of individual daptomycin clearance values versus covariate (creatinine clearance according to Cockroft-Gault formula – C-G CrCL, estimated glomerular filtration rate according to CKD-EPI 2012 and 2021 formulas – eGFR CKD-EPI_2012_ and eGFR CKD-EPI_2021_) plots.

*Orange curves represent smoothing splines. Regression lines are in red.*


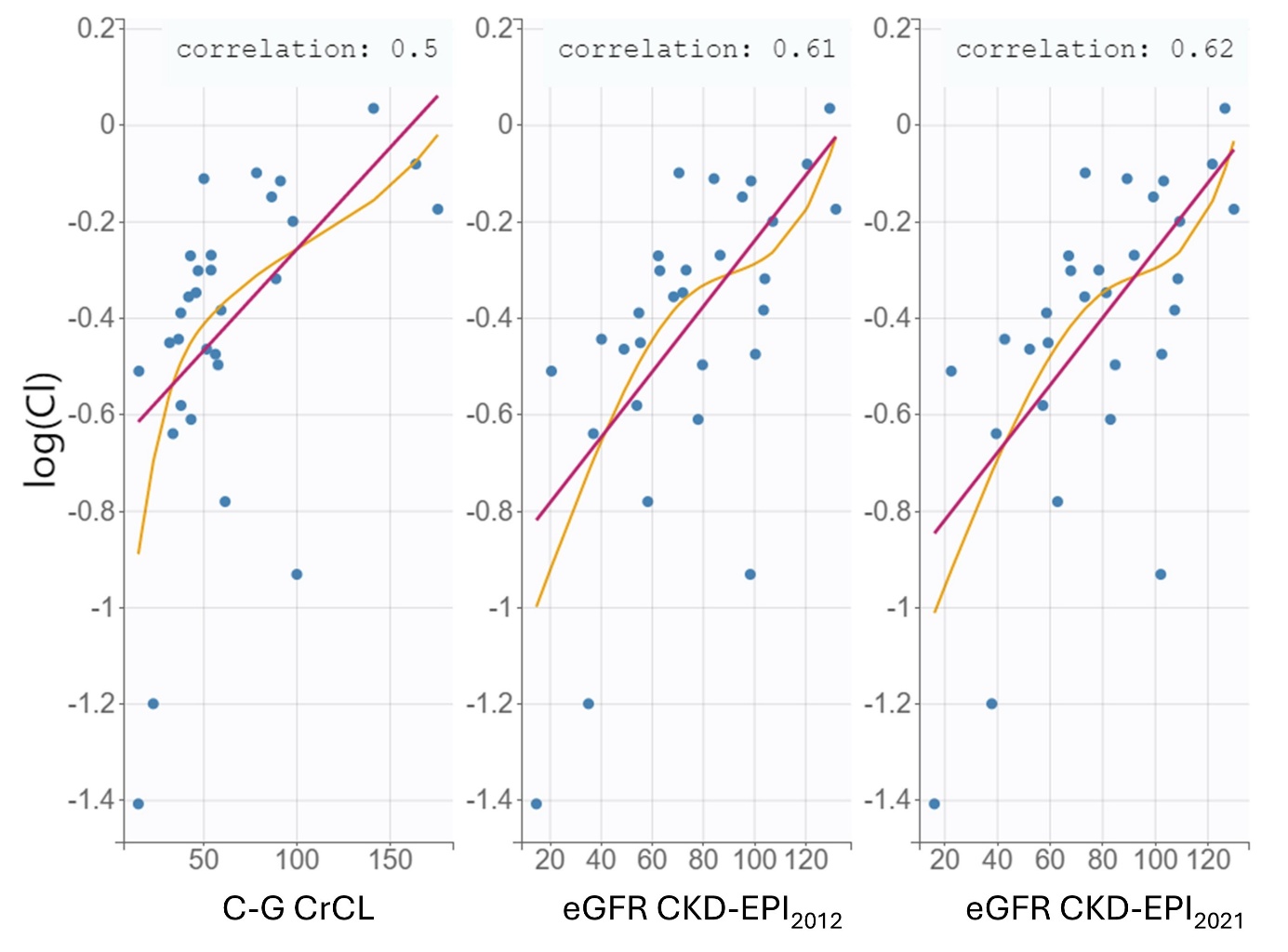


**Figure S2.** The individual-to-population estimates of daptomycin clearance (CL) versus glomerular filtration rate estimated according to CKD-EPI_2021_ formula (eGFR) for both the base structural model and the final covariate model.

**
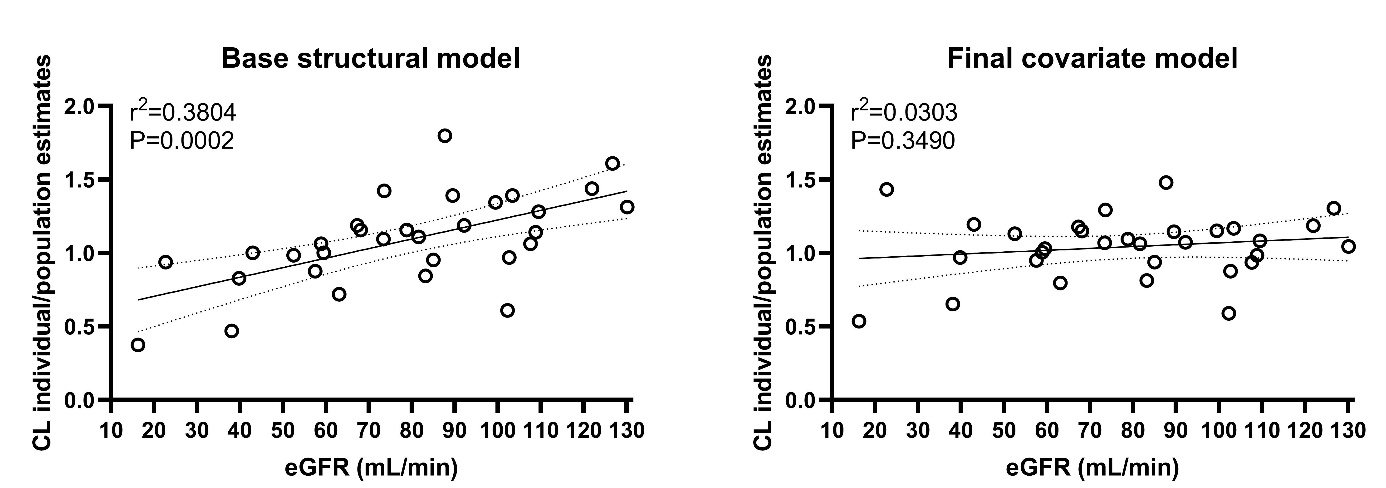
**

**Figure S3.** Goodness-of fit plots obtained from the final daptomycin population pharmacokinetic model: population and individual predictions of daptomycin versus observed concentrations.

*Green points – patients with eGFR ˂ 60 mL/min; blue points - patients with eGFR of 60-90 mL/min; red points - patients with eGFR ˃ 90 mL/min.*

*Orange curves represent smoothing splines.*


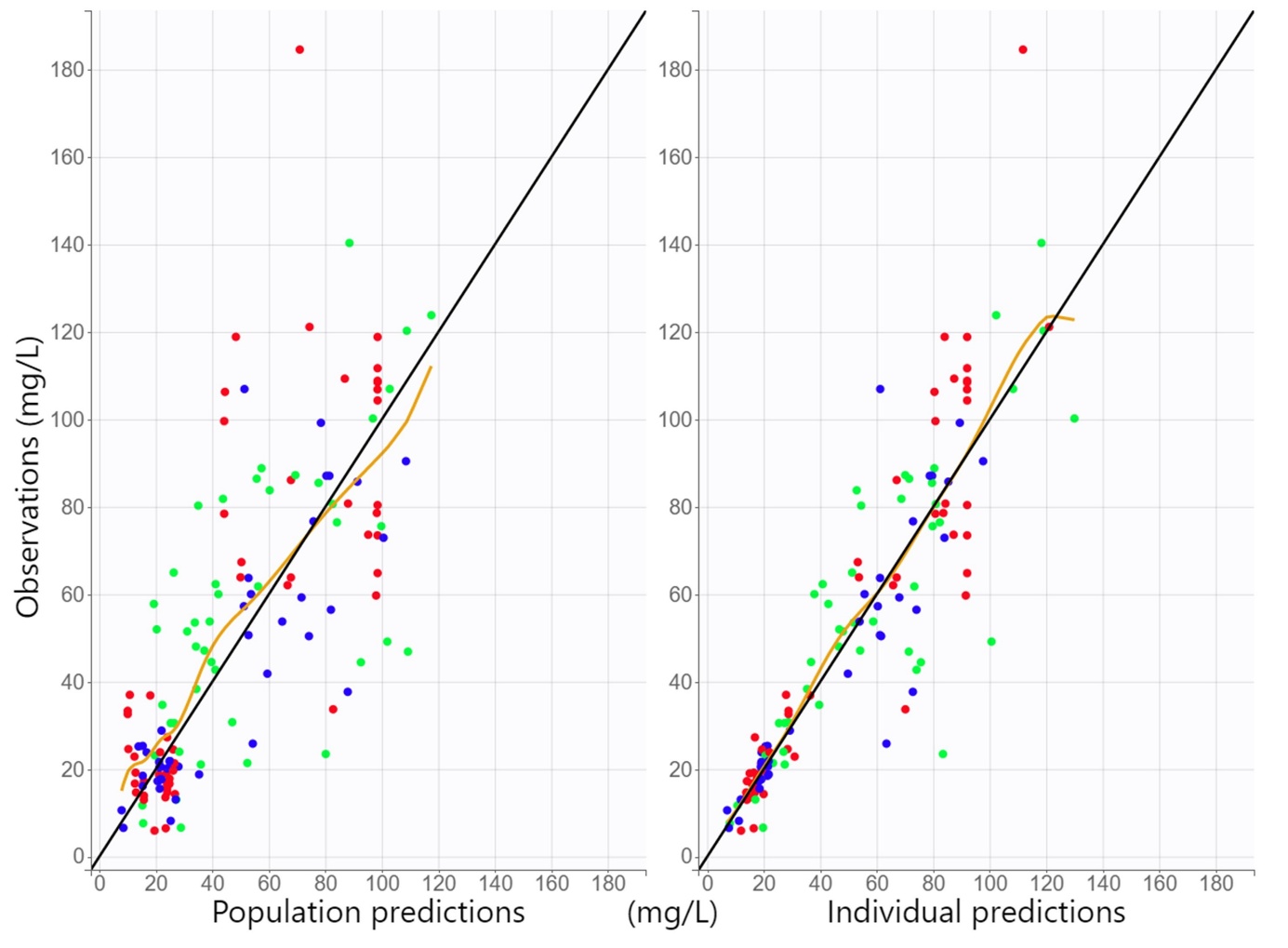


**Figure S4.** Visual predictive check (shaded areas) for daptomycin serum concentration versus time after the last dose for the final model.

*Solid blue lines represent the 10^th^, 50^th^, and 90^th^ percentiles of the observed data. Shaded regions represent 90% confidence interval around the 10^th^ (below blue region), 50^th^ (pink region), and 90^th^ (above blue region) percentiles of the simulated data (dotted black lines). Red areas indicated outliers.*

*
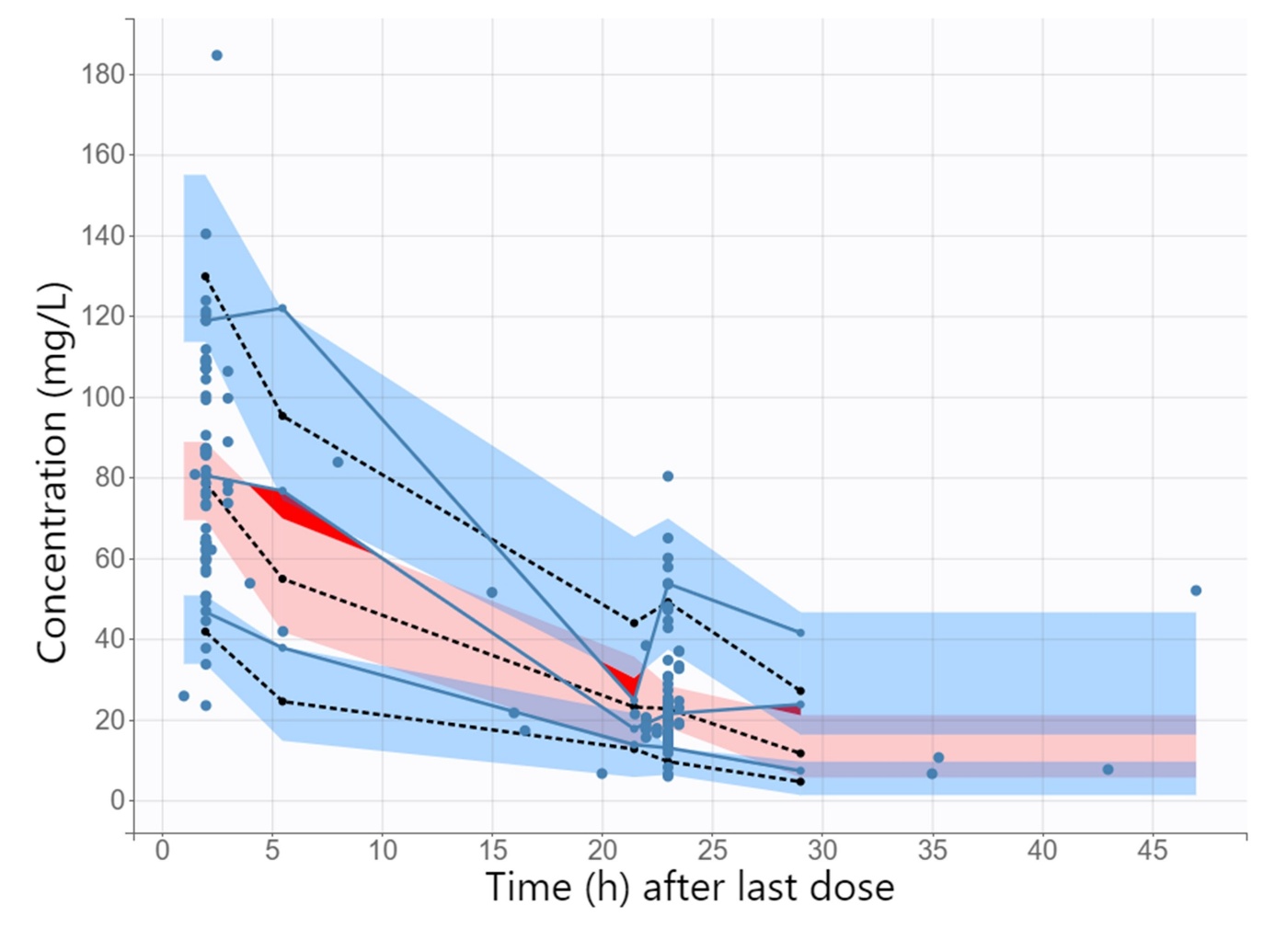
*

**Figure S5.** Visual predictive check (shaded areas) for daptomycin serum concentration versus glomerular filtration rate estimated according to the CKD-EPI_2021_ for the final model.

*Solid blue lines represent the 10^th^, 50^th^, and 90^th^ percentiles of the observed data. Shaded regions represent 90% confidence interval around the 10^th^ (below blue region), 50^th^ (pink region), and 90^th^ (above blue region) percentiles of the simulated data (dotted black lines). Red areas indicated outliers.*


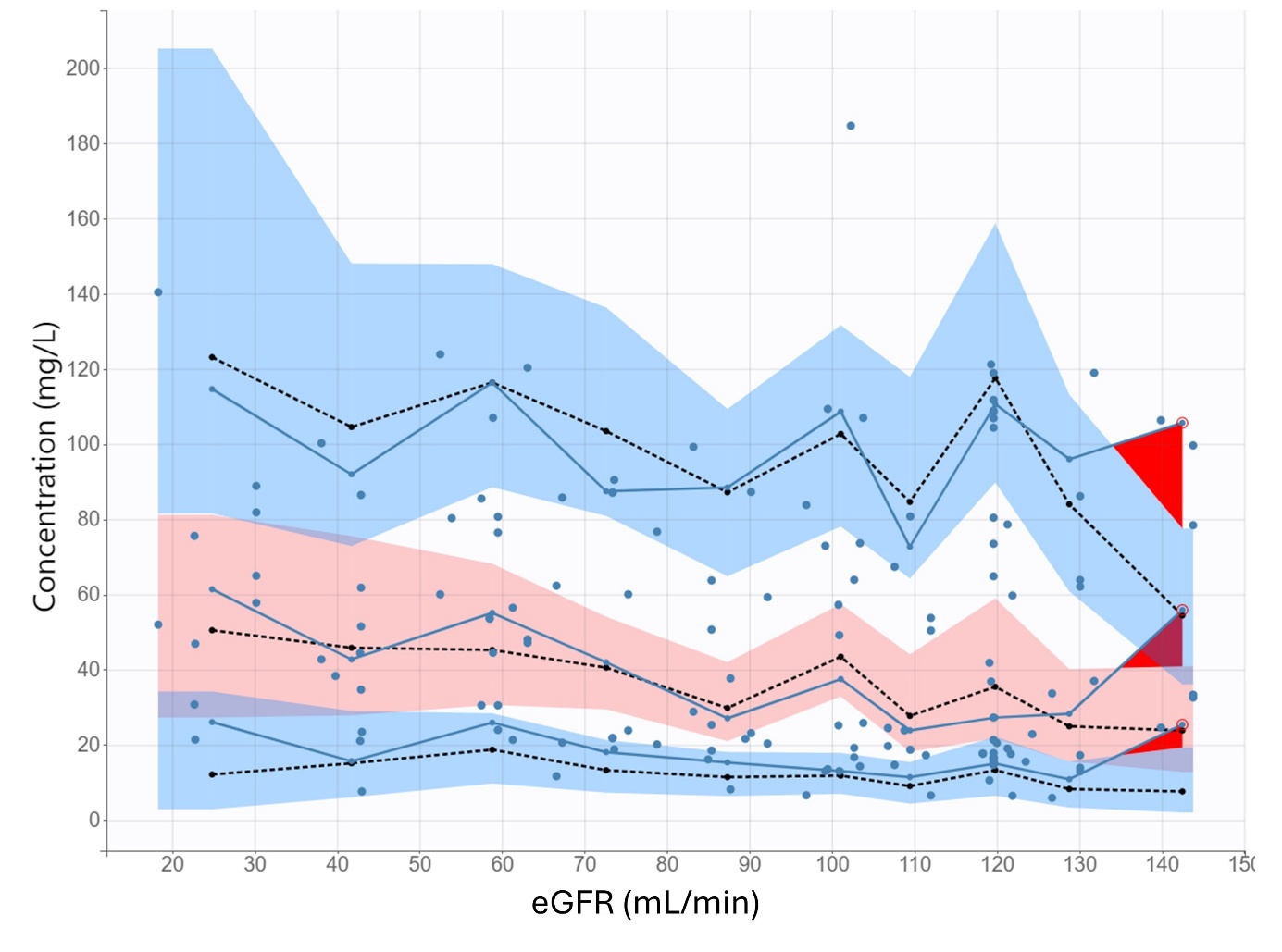

Supplement: Supplemental material — Table S1; Fig. S1 to S5. [file aac.01532-25-s0001.docx]
